# Nutritional Support for Gastrointestinal Cancer Patients: New (and Old) Frontiers in Management, a Narrative Review

**DOI:** 10.3390/nu17243917

**Published:** 2025-12-14

**Authors:** Nazanin Khajoueinejad, Christina Santiago, Kea Turner, Jose M. Pimiento

**Affiliations:** 1 Department of Gastrointestinal Surgery, Moffitt Cancer Center and Research Institute, Tampa, FL 33612, USA; 2 Department of Nutrition Therapy, Moffitt Cancer Center and Research Institute, Tampa, FL 33612, USA; 3 Division of Health Systems, Policy, and Innovation, The University of North Carolina at Chapel Hill School of Nursing, Chapel Hill, NC 27599, USA; kea.turner@unc.edu; 4 Lineberger Comprehensive Cancer Center, The University of North Carolina at Chapel Hill, Chapel Hill, NC 27599, USA

**Keywords:** malnutrition, gastrointestinal cancer, dietary modifications, nutritional counseling, tumor cachexia, nutritional rehabilitation

## Abstract

Malnutrition in patients with gastrointestinal (GI) cancers can be the result of functional and/or anatomical changes in the alimentary tract, secondary to malignancy or oncologic therapies. Understanding the underlying mechanisms of malnutrition in these patients is imperative in providing appropriate interventions that can not only improve quality of life for these individuals, but also improve their tolerance of oncologic treatment and progression towards remission or cure. In this narrative review, we address common nutritional deficiencies associated with GI malignancies, including pancreatic, biliary, and hepatic cancers. Furthermore, we address common issues related to these deficiencies and causes of nutrition barriers as they relate to organ malfunction or surgical alterations of anatomy. Recommendations for counseling, dietary modifications, nutritional supplements, and pharmacologic interventions are provided based on individual barriers and the vital role of multidisciplinary care is highlighted. Additionally, we highlight novel techniques, such as the role of psychosocial care, prehabilitation, digital health, and machine learning, which can improve nutritional outcomes, provide patient-directed care, and improve risk stratification for this complex and multifaceted issue that faces patients diagnosed with GI cancers.

## 1. Introduction

Gastrointestinal (GI) cancers, taken as a whole, correspond to the second cause of cancer death in the United States [1,2]. These malignancies include esophagus, gastric, liver, bile duct, small bowel, pancreas, colon, rectal, and anal cancer [1]. The incidence and prevalence of malnutrition depend on the site of cancer and the anatomic and metabolic alterations that result from it (Table 1).

As has been extensively described, GI malignancies can affect the usual alimentary processes [8,9], alterations of the usual central regulation systems of satiety and hunger [10], nutrient intake, digestion, intestinal transit, absorption, and waste elimination [8,9,11,12]. Anticancer therapies used for the treatment of these malignancies are associated with multiple side effects that further impact the patient’s nutritional status [8,13]. This is true for chemotherapy, immunotherapy, cell therapies, radiation, and surgery [8]. Conversely, the nutritional status significantly impacts outcomes for cancer treatment [14,15,16,17].

In this narrative, scoping review, we highlight the most common micro- and macronutrient alterations impacting gastrointestinal cancer patients, provide recommendations for therapeutic clinical strategies for addressing these alterations, and discuss novel pharmaceutical and interventional approaches to identifying patients who are at risk of malnutrition.

## 2. Methods

PubMed, Medline, and Cochrane Review databases were queried for randomized clinical trials, meta-analyses, and systematic reviews focusing on micronutrient and macronutrient deficiencies in patients with gastrointestinal malignancies, management of symptoms, and clinical and pharmaceutical interventions. Similarly, the same approach was utilized to identify novel approaches to addressing nutritional deficits in cancer patients.

## 3. Nutritional Challenges Based on Cancer Site and Cancer Treatment

Individuals diagnosed with GI cancers can experience a myriad of nutritional challenges. Jacobson et al. provide a taxonomy of the long-term nutrition challenges associated with operations for GI cancers, based on the anatomic (restrictive or malabsorptive) or functional consequences of such procedures [8]. This taxonomy is a useful framework for the acute nutritional challenges associated with tumors located in the GI tract, based on their location and organ involvement.

In this context, tumors can cause anatomic alterations, such as mechanical complete or partial obstruction, functional impairment (i.e.,; pancreatic malabsorption), and what one can call central or systemic alterations (i.e.,; anorexia, cancer cachexia, or diabetes) [18,19,20]. Similarly, antineoplastic treatment can cause or worsen these alterations [21,22,23,24]. The following table (Table 2) presents common nutrition challenges and impactful symptoms for the five most prevalent GI cancers by anatomic location: pancreatic, gastroesophageal, colorectal, and liver cancers [25].

## 4. Therapeutic Strategies

### 4.1. Specialized Evaluation

Throughout their oncologic journey, patients diagnosed with GI cancers can present with increased nutrient requirements. Additionally, oncologic therapies present a host of nutritional barriers that are specific not just to the type of treatment, but to each individual patient [8,35,36]. Therefore, specialized evaluation by a registered dietitian (RD) is a critical step in addressing these barriers to maintaining appropriate nutrition during initial diagnosis, treatment, and surveillance. An RD can perform a comprehensive nutritional evaluation and can develop individualized nutrition plans to address intake requirements while managing nutrition impact symptoms [37,38]. The following basic recommendations are part of the national and international guidelines [18,35,36,37]:

- Adult oncology patients should undergo regular malnutrition risk screening and nutrition assessment should be completed by an RD.
- RD evaluation and personalized treatment plans for GI cancer patients can help improve nutrition-related outcomes.
- Continued RD assessment is required, as treatment side effects and cancer symptoms may change over time and nutrition recommendations should be adjusted as needed to improve patients’ outcomes [18].
- Incorporating other specialties, such as case management, social work, physical therapy, and genetics, among others, is necessary for completing a comprehensive evaluation and developing a management plan to address factors such as patients’ access to food, patients’ support system or lack thereof, individual dietary preferences, genetic concerns, and physical limitations, including challenges with activities of daily living.

### 4.2. Identifications of Barriers to Nutrition and Recommended Dietary Modifications

Patients with GI cancers may require nutrition guidance from RDs and dietary modifications to minimize the effects of nutrition-impact symptoms and other nutrition challenges (e.g., malabsorption) on nutrient intake, digestion, and absorption. Below, we summarize the most common nutrition barriers experienced by GI cancer patients and the recommended approaches for nutrition counseling and dietary modifications (Table 3).

- Malabsorption/essential fatty acid deficiency [39]
  ○ Patients who have undergone significant bowel resection, those on long term fat-restricted diets due to chyle leaks, or those who have pancreatic insufficiency are at risk of essential fatty acid deficiency [40]. Physical manifestations include a dry, scaly rash, hair loss, and poor wound healing [39]. Identifying the underlying cause is important for developing an appropriate nutrition plan.
  ○ Providing at least 10% of total calories from fat prevents the development of this alteration [39].
  ○ For patients with a fat-free diet, assuring supplementation of essential fatty acids is necessary.
- Exocrine pancreatic insufficiency (EPI)
  ○ EPI is frequently seen in patients with chronic pancreatitis with atrophy, pancreatic duct obstruction, bypass surgery, or after major pancreatic surgeries. EPI may occur due to a decrease in exocrine cells to secrete digestive enzymes, inadequate bicarbonate production causing inactivation of enzymes by stomach acid, or a mismatch between enzyme release and chyme transit. Inadequate fat digestion can limit the absorption of fat-soluble vitamins A, D, E, and K, as well as influence malnutrition, osteopenia, and sarcopenia [40].
  ○ Many EPI symptoms are heterogeneous and common with oncological treatment. Regular screening for at-risk patients and comparing symptoms with a diet recall can help identify EPI. A fecal elastase test or qualitative fecal fat test can provide insight. However, these tests may not identify EPI in patients with adequate enzyme production and mismatched timing with chyme.
  ○ Weight-based dosing of pancreatic enzyme replacement is the treatment of choice and resources online can help tailor personalized plans [28,40,41].
- Poor appetite/anorexia [42,43,44]
  ○ Poor appetite is routinely reported as a major side effect from cancer treatment and risk factors include chemotherapy and surgery.
  ○ Anorexia can be multifactorial: contributing factors include tumor-related inflammatory markers and side effects of oncology treatment (i.e., chemotherapy, targeted therapies, immunotherapy and radiation).
  ○ Alterations in the mood (i.e., depression and anxiety) related to cancer diagnosis can also cause suppression of the appetite [45]. Pharmacological agents such as progesterone analogs, corticosteroids, and antipsychotics may provide appetite stimulation. There is also new evidence to suggest that targeted therapy for stress-associated cytokines can provide benefits for weight gain [46].
- Dysgeusia/hypogeusia [47,48]
  ○ Taste changes are very individualistic and predominantly occur with chemotherapy or major surgical interventions such as cytoreductive surgery, pancreatic resection, extensive colorectal resections, or gastroesophageal interventions.
  ○ Patients can experience a lack of taste, over-accentuated taste, or “off” flavors. Patients may prefer salty or sweet flavors or find that food has no flavor. Management involves an assessment of current acceptable flavors and individualized nutrition recommendations.
- Nausea/vomiting [49,50]
  ○ Appropriate use of anti-emetics and diet modification can help manage symptoms.
  ○ Foods that are warm, greasy, or high in fiber might exacerbate nausea.
  ○ Specific algorithms for the pharmacologic management of nausea and vomiting are found in the societal guidelines for nutritional support of cancer patients [49,50].
- Diarrhea [51,52]
  ○ Diarrhea can be seen with chemotherapy, radiation therapy, immunotherapy, and following surgical resection of various parts of the GI tract. Excessive fluid losses may lead to renal injury, hypotension, and cardiovascular concerns. Pharmacologic approaches, along with dietary management, can lessen symptoms and improve quality of life (QOL).
  ○ An accurate picture of the frequency and volume of stooling should be obtained to evaluate treatment effectiveness.
  ○ Temporary lactose intolerance can be seen with many chemotherapy agents [53], including 5-FU regimens, and may be reversed upon discontinuation of treatment. Short-term avoidance or choosing low-lactose products can improve symptoms.
  ○ Etiology of diarrhea can be vast and may be a direct result of treatment or due to side effects from treatment. Identifying the cause of diarrhea can help to target management of symptoms.
  ○ Intake of oral rehydration solutions can improve fluid absorption in the setting of diarrhea. Patients should be instructed to sip electrolyte-balanced fluids throughout the day.
- Delayed gastric emptying [8]
  ○ Delayed gastric emptying may be seen in oncology patients with diabetes mellitus or following pancreatic, gastric, or esophageal resections.
  ○ Symptoms may include nausea, vomiting, bloating, early satiety, or abdominal pain.
  ○ Dietitians should monitor weight changes in addition to dietary history to identify eating habits that may exacerbate symptoms of delayed emptying. This should include a review of symptoms, portion sizes of food and beverages, meal frequency, timing of food and fluids, and use of any supplements that may lead to delayed emptying.
  ○ Prokinetics have been used for its management [54,55], but careful evaluation for mechanical bowel obstruction before initiating therapy is necessary.
- Dumping syndrome [8,56]
  ○ Dumping syndrome can be identified as early dumping or late dumping [8]. Early dumping syndrome is seen 10–30 min after eating and occurs when hyperosmolar chyme flows through the bowel; this results in excess luminal fluid secretion, which minimizes the osmolarity of chyme. This is followed by rapid intestinal transit that leads to symptoms which include diarrhea, bloating, nausea, abdominal pain, dehydration, and vasomotor symptoms. Late dumping syndrome occurs 1–3 h after eating. Symptoms are often due to excess insulin secretion from the rapid transit of concentrated carbohydrates through the small bowel. Symptomatic hypoglycemia is characterized by flushing, dizziness, heart palpitations, and sudden fatigue.
  ○ Timely identification and intervention of dumping syndrome can alleviate discomfort and fear around eating.
  ○ There are specific diagnostic criteria and algorithmic management strategies for this entity [56].
- Risk of bowel obstruction
  ○ Intestinal strictures can occur from adhesions, bowel ischemia, or tumor proliferation and can increase the risk of bowel obstructions.
  ○ Significant adhesions or recurrent obstructions indicate a need for a stricter low-fiber diet.
  ○ Patients may overly restrict their diet out of fear of symptom recurrence, which may lead to malnutrition. Proper assessment and intervention by a registered dietitian can inform adequate oral intake.
- Micronutrient deficiency
  ○ Micronutrients are absorbed in various locations throughout the gastrointestinal tract, and deficiencies may be related to prior surgical resection, poor oral intake, or malabsorption syndromes [8,57].
  ○ The dietitian should review typical intake and assessment of dietary quality.
  ○ Supplementation of Vitamin B12 is recommended for patients after total gastrectomy or total colectomy. This can be supplemented orally at a high enough dose to achieve similar results when compared to parenteral supplementation [58,59].
  ○ Iron deficiency can be seen in patients with small bowel resections involving the duodenum and proximal jejunum, which are primary sites for iron absorption [60]. As such, patients who have undergone a Roux-en-Y reconstruction after resections are at increased risk of iron deficiency anemia. Additionally, the loss of gastric acid after a partial or total gastrectomy may limit the bioavailability of iron for absorption [60].
  ○ Following an esophagectomy, patients are at increased risk of vitamin B12, vitamin D, and iron deficiency, and these deficiencies may persist for up to 24 months [61,62].
  ○ Calcium is absorbed throughout the small intestine, with active absorption primarily occurring in the duodenum and proximal jejunum and passive absorption seen primarily in the jejunum [63]. Surgical resections, including ileostomy formation, Whipple, esophagectomy, and total gastrectomy, may affect calcium absorption. Vitamin D deficiency may also influence calcium deficiency, as vitamin D is involved in the intracellular transit of calcium.
  ○ Research demonstrates an association between higher calcium intake and reduced incidences of colorectal cancer [64]. Moreover, vitamin D supplementation has been shown to improve survival in cancer patients [65] and specifically in colorectal cancer patients [66]. There is very limited high-quality data on micronutrient needs in patients with cancer diagnosis and undergoing oncologic management.

### 4.3. Medical Nutrition Interventions

Evidence-based approaches to decreasing the development of malnutrition during the different periods of the continuum of cancer care have been published [18,36,37]. They include some of the aforementioned dietary-based interventions to address the common challenges and symptoms associated with cancer and its therapies [36]. The European Society for Clinical Nutrition and Metabolism (ESPEN) has performed a very thorough review of the evidence for medical nutrition recommendations for cancer patients, using the GRADE method to achieve the recommendation level based on strength or recommendation (weak to strong), level of evidence (low to high), and degree of consensus, with a strong consensus denoted when reaching at least 80% of the panel. The following table (Table 4) summarizes the medical therapy recommendations for cancer patients [36].

## 5. Pharmacological Interventions

An important factor of malnutrition in cancer patients is anorexia and cachexia, which occur both as a result of treatment side effects and central/systemic alterations related to the underlying malignancy. Counteracting these syndromes can reduce the burden of malnutrition in GI cancer patients.

Intense interest in pharmacological agents to address anorexia and cachexia in cancer patients has been commonplace, with various medications to stimulate appetite and offset anorexia associated with underlying malignancies or with the treatment side effects of oncologic therapies having been studied in preclinical or small clinical settings [42,43,44,67]. Until recently, only two medications were recommended for utilization for appetite stimulation in cancer patients. Recent advances in understanding the physiopathology of anorexia and cachexia highlight a potential role for the use of antipsychotics and targeted monoclonal antibodies to combat cancer-related cachexia, which inevitably leads to malnutrition [53,68].

### 5.1. Historically Used Mediations: Megestrol and Corticosteroids

Until recently, megestrol and corticosteroids were the most common medications used to address appetite loss in cancer patients.

Megestrol, a synthetic progestogen, was first introduced for the treatment of hormonal-associated cancers and was observed to demonstrate a side effect of weight gain in these treated patients [69]; as such, it was then studied as a potential pharmacological tool for the alleviation of poor appetite in cancer patients in the late 1980s [69,70]. To date, multiple randomized controlled trials and meta-analyses have evaluated the benefit of progesterone analogs for the management of cachexia and have shown improvement in appetite and quality of life [71,72,73,74]. The benefits of megestrol, however, must be weighed against its known complications. Patients treated with megestrol are at higher risk of developing dyspnea, peripheral edema, thromboembolic events, and potentially death. These side effects raise concerns for prolonged use of this hormonal therapy [67]. Based on these results, the most recent American Society of Clinical Oncology (ASCO) guidelines provide moderate strength recommendations in favor of the short-term use of progesterone analogs [71].

In a landmark randomized double blinded study of patients with advanced cancer published in 1974, patients who received dexamethasone reported an improvement in their appetite [75]. The benefit for appetite stimulation was further supported by a systemic review of multiple randomized controlled studies [67]. Despite the benefit to appetite stimulation, the ideal dosing, timing, and duration of the treatment is still not clearly defined. Even more, the benefit to weight gain has not been consistently demonstrated. Based on these results, the ASCO guidelines on the management of cancer cachexia support a short-term course of this medication in cancer patients for appetite stimulation [71].

While megestrol and dexamethasone remain the two most used medications for appetite stimulation in cancer patients, the two had not been compared head-to-head until recently. A phase III double blinded clinical trial comparing megestrol, dexamethasone, and a placebo daily did not demonstrate a statistically significant difference in appetite scores between the three study arms. Of note, the study was only able to evaluate one week of data, given its failure to increase appetite scores more than 25% from the baseline [76]. With this new data and existing historical evidence, the use of megestrol and steroids come into question.

### 5.2. Antipsychotic Medications: Olanzapine

The use of olanzapine for treatment of psychiatric disorders was associated with unexpected weight gain. This side effect has led to the utilization of the anti-psychotic for use in patients with anorexia nervosa [77]. Additionally, a short course of treatment is also used to treat chemotherapy-related nausea in cancer patients [77,78].

Recently, a randomized double-blinded placebo-controlled study of olanzapine in patients with chemotherapy-related anorexia and diagnosis of locally advanced or metastatic gastric, HPB, or lung cancer demonstrated a statistically significant improvement in weight gain in the study arm. Patients in the study received olanzapine 2.5 mg daily for 12 weeks, with the first dose being received on the first day of systemic therapy. Patients receiving Olanzapine demonstrated a significantly decreased incidence of severe malnutrition at study completion (14% vs. 39%; *p*: 0.01), and significant weight gain compared to the placebo (60% vs. 9%; *p* < 0.001). The study arm also demonstrated improvements in appetite at 12 weeks, improved nutrition scores, higher daily calories, and overall, minimal adverse events were demonstrated [79].

Based on the promising results of this study, the ASCO guidelines for the management of cancer cachexia were updated in 2023 to recommend the use of low-dose olanzapine for addressing poor appetite and improving weight gain in patients with advanced cancers [46,71]. A phase III clinical trial is currently recruiting patients to compare the effects of olanzapine to megestrol in addressing appetite loss (NCT04939090).

### 5.3. Targeted Therapy—Ponsegromab

Targeted therapies against molecules that play a role in cancer-related cachexia and malnutrition hold a promising future in the treatment of cancer patients [80]. A recent phase II study has demonstrated promising results for the blockage of a stress-induced cytokine, Growth differentiation factor 15 (GDF-15), with a humanized monoclonal antibody [68,80,81].

Growth differentiation factor 15 is a stress-induced cytokine that has demonstrated a correlation with weight loss and loss of skeletal muscle mass [80,81]. Ponsegromab is a highly selective humanized monoclonal antibody that inhibits GDF-15 in circulation and has demonstrated reduction in weight loss and skeletal muscle loss in translational models of cancer-related cachexia [68]. A phase-2 randomized double blinded placebo-controlled trial compared three doses of the study drug to placebo [68]. More than half of the participants in this study were diagnosed with GI cancers, including pancreatic and colorectal cancers (61%). Patients in the study arm experienced a dose-dependent increase in their weight compared to the placebo at 12 weeks. Specifically, patients in the highest dose group, 400 mg, gained back more than 5% of their body weight [68]. Beyond these objective improvements, subjects reported improved appetite and physical activity. This outcome was consistent across different cancer types, and exposure to platinum chemotherapy, as well as the baseline inflammatory state, as evaluated by CRP and albumin [68]. The results of this trial demonstrate a promising future for more targeted therapies in addressing the complex physiopathology of cancer-related cachexia and malnutrition [81].

## 6. Future Directions in Addressing Malnutrition in Cancer Patients

### 6.1. Prehabilitation—The Need for Standardization

Prehabilitation programs that provide nutrition support as a standalone intervention or multi-modal support (e.g., nutrition and exercise) have demonstrated effectiveness for improving nutritional outcomes for GI cancer patients preparing for surgery. Prehabilitation can improve nutritional status (e.g., PG-SGA score) and dietary intake and minimize losses in weight and body mass [82,83,84]. These interventions may have the greatest impact on patients with moderate or severe malnutrition at baseline [85]. Prehabilitation programs can also lead to improvements in functional capacity and quality of life for GI cancer patients receiving surgery [84,86], outcomes highly correlated with nutritional outcomes. Despite the promising evidence base for nutrition prehabilitation, there are notable research gaps that need to be addressed [87].

Across nutrition prehabilitation studies, the ‘nutrition’ intervention component is often poorly defined [88], making it difficult to discern which nutrition interventions lead to improvements in patient outcomes. Nutritional prehabilitation could include a combination of nutrition interventions, such as dietary counseling, patient education, oral nutrition supplements, immunonutrition, and energy intake monitoring [88]. Many of these interventions have failed to measure the impact of the intervention on nutrition outcomes or failed to use validated malnutrition assessments [88]. A recent review found that most prehabilitation studies in oncology (two-thirds) do not measure nutritional outcomes [88]. When nutritional outcomes are measured, there is heterogeneity in what is measured [87] and a limited study of long-term outcomes, such as survival. All these factors highlight the need for standardizing how nutrition prehabilitation interventions are defined and what outcomes are measured.

Research is also needed to examine the delivery and implementation of nutrition prehabilitation programs for GI cancer patients. Given how many components can be included in nutrition prehabilitation, there is a need for comparative effectiveness research to determine which components are most effective at improving patient outcomes. Second, there is a need for intervention delivery research to determine the optimal timing, dosage, and duration of nutritional interventions. For example, studies suggest that nutrition counseling delivered before or at the start of cancer treatment may be more effective at improving nutritional outcomes compared with nutrition counseling that is provided after treatment is already well underway [89,90,91,92]. Well-designed clinical trials are needed to definitively establish the ideal timing of nutrition prehabilitation interventions, how much intervention is needed, and for how long.

Studies suggest that adherence to nutrition prehabilitation varies considerably across studies and is inconsistently measured and reported [33,87,93,94,95]. Studies report several patient barriers that may affect adherence to nutrition prehabilitation. Patients’ physical or mental health may make it difficult to participate in nutrition prehabilitation (e.g., symptom burden, mobility, cognition, depression, anxiety) [96,97]. Patients and their caregivers may also lack awareness about nutrition prehabilitation [97], or have difficulty with logistics (e.g., scheduling, time commitment) [96]. Social determinants of health (e.g., transportation barriers, food insecurity, financial hardship) [98,99] can also affect participation. Similarly, healthcare settings may vary in their capacity to provide nutrition prehabilitation due to insufficient dietitian staffing (e.g., patient-to-dietitian ratios), availability of dietitians with specialized expertise in GI cancer, and inadequate insurance reimbursement for oncology nutrition services [41,100,101]. Innovation is needed to determine what strategies can be used to reduce patient barriers to nutrition prehabilitation and promote equitable access to nutrition prehabilitation across settings.

### 6.2. Behavioral Science and Role of Psychosocial Care

Behavioral science is increasingly being applied to cancer prehabilitation research to optimize patient engagement and overcome barriers to patient participation and adherence [102]. Recent studies have used behavior change techniques such as self-monitoring, goal setting, social support, and motivational messaging to support the behavior changes that are required in prehabilitation programs [102,103,104,105,106,107]. Because the application of behavioral science to cancer prehabilitation is still limited, there is limited evidence on what behavioral change techniques may be most effective in prehabilitation, how behavior change should be sequenced (e.g., implementing multiple changes simultaneously or sequentially), or what approaches are most effective at promoting long-term behavior change beyond prehabilitation [102].

Psychosocial care has been integrated into cancer prehabilitation to support patient behavior changes. Prehabilitation programs have included mindfulness, stress management, cognitive-behavioral strategies, resilience training, and return-to-work support [108,109,110,111,112]. Integration of psychosocial care into prehabilitation can improve patient outcomes, such as anxiety, depression, and fatigue [109,110,111], determinants that can also affect nutrition. Prior reviews suggest that psychosocial care may be more commonly integrated into prehabilitation for other cancer types (e.g., gynecological cancers) and less commonly used in prehabilitation programs for GI cancer [113,114,115]. Given that individuals with GI cancer experience higher rates of anxiety, depression, and fatigue, compared with many other cancer types [116,117], there is a need to test the integration of psychosocial care into GI prehabilitation programs. Researchers could evaluate, for example, if there is a synergistic effect between psychosocial care and nutrition care that leads to improved patient outcomes.

### 6.3. Digital Health

Digital health is being tested as a strategy to improve access to and the delivery of nutrition prehabilitation. Studies have tested telemedicine as a method for delivering nutrition counseling before surgery, either through telephone or videoconference. Telemedicine-delivered nutrition prehabilitation has demonstrated feasibility and acceptability and improved access to nutrition care for certain GI cancer patients (e.g., rural residents) [61,118,119,120]. Tele-prehabilitation has also demonstrated a preliminary effect for improving nutritional outcomes, such as nutritional status, dietary intake, and weight maintenance, and related outcomes (e.g., quality of life) [121,122] in GI cancer, but the available evidence is limited to small pilot studies. Additionally, flexibility in delivery mode can improve patient adherence and satisfaction with prehabilitation programs [94,102,123,124].

Digital health technologies, such as mobile apps, web-based platforms, and wearable devices, are being tested to support nutrition prehabilitation delivery. For example, studies have used mobile apps, web-based platforms, and wearable devices to deliver nutrition education, track dietary intake, and assess nutritional status and symptoms, and preliminary studies suggest that these technologies are feasible [118,125,126,127,128]. Digital technology utilization in addressing nutritional prehabilitation holds a promising future but work is necessary to (1) establish the impact of digital health technologies on nutritional outcomes; (2) determine which strategies are most effective at overcoming barriers to technology use; (3) address the usability issues of existing technologies through human-centered and human factors research; and (4) test digital technologies in real-world settings and populations to gather evidence on implementation.

### 6.4. Machine Learning

Commonly used nutritional assessment tools and surveys are often limited by their subjective nature and poor performance, capturing the complex and multifaceted nature of cancer-related malnutrition and cachexia [129]. Machine learning models introduce a new frontier that allows for incorporation of clinically available data, including molecular markers, patient-reported symptoms, and even imaging to predict the presence of malnutrition and cancer-related cachexia and, in doing so, introduces the opportunity for more prompt and patient-tailored interventions [130,131,132,133].

Deep machine learning algorithms have demonstrated an enhanced predicting ability to correctly identify malnutrition in patients with gastric cancer when utilizing imaging features such as psoas muscle at the third lumbar vertebrate and clinical prognostic features such as BMI and albumin and lymphocyte count [134]. Large retrospective studies support the use of machine learning in predicting the presence of cachexia in a broad range of cancer diagnoses, even when the electronic health record (EHR) lacks information about weight loss [132,133]. This finding is very appealing, as weight loss is one of the most commonly used tools in identifying patients at risk of developing cachexia and the implementation of machine learning models has demonstrated the ability to accurately predict cachexia in cancer patients, even when this vital measurement is absent [132,133]. These models take advantage of other important clinical factors, such as changes in appetite, clinical lab values, and changes in muscle mass to predict malnutrition. This innovative approach can serve as a vital tool to identify at-risk individuals who otherwise would not be able to be assessed for malnutrition, given the missing clinical data points [132,133].

Perhaps the more critical application of machine learning models is for the identification of patients at risk of progression to developing malnutrition or cancer-related cachexia [131,135]. Retrospective data have demonstrated a promising ability to utilize machine learning to identify patients with pre-cachexia with high accuracy. Furthermore, it provides a valuable tool for more individualized risk stratification of patients that may not be feasible with the existing clinical screening tools [131]. This important prognostication tool can allow for more individualized goal-directed care, intervention, and follow up.

## 7. Conclusions

Nutrition complications in patients with gastrointestinal cancers are a serious and common problem; accurate diagnosis of the nutritional challenge and associated symptomatology is paramount for developing a personalized dietary recommendation. Novel technologies will help in identifying patients who are at risk of developing these devastating problems. A comprehensive multidisciplinary evaluation is necessary early and often to address these complications; therapeutic pharmacological advances and technological innovations in delivery of care should pave the way to decrease the incidence and clinical impact of malnutrition on gastrointestinal cancer patients.

## Figures and Tables

**Table 1:** Reported incidence of malnutrition per cancer type.

Cancer Location	Incidence of Malnutrition	References
Esophageal	22–73%	[3,4,5]
Gastric	10–50%	[6]
Pancreatic	9–52%	[5,6]
Liver	7–20%	[5]
Colorectal	23%	[7]

**Table 2:** Nutritional challenges and various nutrition impact symptoms that patients may experience, based on cancer site.

Disease Site	Nutrition Challenges	Nutrition Impact Symptoms
**Pancreatic** [26,27,28]	Exocrine insufficiency Fat-soluble vitamin deficiencies Protein and micronutrient deficiencies Weight loss, sarcopenia, cachexia Bone health complications Gastric outlet obstruction	Fatty stools (steatorrhea) Chronic diarrhea Abdominal pain and bloating Feeling full early (early satiety) Fatigue Loss of appetite (anorexia) Nausea Constipation Distress Cold dysesthesia
**Esophageal and Gastric** [8,29,30,31]	Esophageal obstruction Gastric outlet obstruction Vitamin deficiencies (folate, B12, iron) Pernicious anemia Malabsorption Dehydration Sarcopenia	Dysphagia Odynophagia Nausea Vomiting Early satiety Abdominal pain and bloating Reflux Anorexia Cold dysesthesia
**Colorectal** [8,32,33]	Malabsorption Diarrhea Iron deficiency anemia Malignant obstruction	Anorexia Abdominal pain and bloating Early satiety Nausea/vomiting Diarrhea Constipation Obstipation Cold dysesthesia
**Liver** [34]	Fat malabsorption in setting of hyperbilirubinemia Sarcopenia Malnutrition related to underlying cirrhosis or fatty liver disease Cachexia Dehydration and electrolyte imbalances in setting of hepatorenal syndrome	Anorexia Abdominal pain, bloating Ascites Early satiety

**Table 3:** Common nutrition barriers in GI cancer patients and recommended nutrition counseling and dietary modifications.

Barrier to Intake	Recommended Nutrition Strategy
Malabsorption—Fatty Acid Deficiency	- Ensure ~10% of total calorie intake from fat. - Encourage nut butters, vegetable oils, nuts, seeds, avocados, and condiments.
Malabsorption—Exocrine Pancreatic Insufficiency	- Weight-based pancreatic enzyme replacement. - If EPI symptoms continue despite appropriate enzyme use, consider a lower-fat diet. - Smaller portions incorporating grains, fruits, and high protein diet.
Poor Appetite	- Follow an eating schedule to not skip meals. - Add in calorie- and protein-dense options to all meals and snacks. - Choose calorie- and protein-containing fluids, as tolerated. - Consider pharmacologist interventions.
Dysgeusia/Hypogeusia	- Identify foods that are palatable or unpleasant. - Trial seasonings or acidic options, as tolerated. - Limit metallic cups or utensils if foods taste metallic.
Nausea/Vomiting	- Choose small, frequent meals that are room temperature or cold. - Consider anti-emetics before oral intake. - Limit greasy, fried, or spicy foods.
Diarrhea	- Choose small, frequent meals. - Sip most fluids between meals. - Include complex carbohydrates with meals (potatoes, rice, pasta, etc.). - Consider a trial of lactose-free dairy. - Limit greasy, fried foods, large volumes of insoluble fibers, and concentrated sweets.
Delayed Emptying	- Eat small, frequent meals and chew foods well. - Limit large portions of high-fiber foods and high-fat foods. - Liquids may be better tolerated than solids later in the day. - Consider enteral nutrition support if unable to increase and maintain consistent oral intake.
Dumping Syndrome	- Include fat and protein foods with all meals and limit simple sugars. - Eat small, frequent meals. - Minimize liquid intake at mealtime.
Risk of Bowel Obstruction	- Eat small, frequent meals and chew foods well. - Limit large portions of fiber-rich foods. - Limit gas-forming foods.

**Table 4:** Medical therapy recommendation in cancer patients modified from the ESPEN guidelines [36].

Topic/Strength of Recommendation	Recommendation	Level of Evidence
Energy requirement/strong	Recommend that total energy expenditure of cancer patients, if not measured individually, be assumed to be similar to healthy subjects and generally range between 25 and 30 kcal/kg/day.	Low/Consensus
Protein requirement/strong	Recommend that protein intake should be above 1 g/kg/day and, if possible, up to 1.5 g/kg/day.	Moderate/Strong Consensus
Efficacy of nutrition intervention/strong	Nutritional intervention to increase oral intake in cancer patients who are able to eat but are malnourished or at risk of malnutrition. This includes dietary advice, the treatment of symptoms and derangements impairing food intake (nutrition impact symptoms), and offering oral nutritional supplements (ONS).	Moderate/Consensus
Specialized diets in cancer patients/strong	Recommend not to use dietary provisions that restrict energy intake in patients with or at risk of malnutrition. Recommendation B3-2; strength of recommendation is strong; level of evidence low; strong consensus.	Low/Strong Consensus
Mode of nutritional therapy when to escalate/strong	If a decision has been made to feed a patient, we recommend enteral nutrition (EN) if oral nutrition remains inadequate despite nutritional interventions (counseling, ONS), and parenteral nutrition (PN) if EN is not sufficient or feasible.	Moderate/Strong Consensus
Refeeding syndrome/strong	If oral food intake has been decreased severely for a prolonged period, we recommend increasing (oral, enteral, or parenteral) nutrition slowly over several days and to take additional precautions to prevent a refeeding syndrome.	Low/Consensus
Home artificial nutrition/strong	In patients with chronic insufficient dietary intake and/or uncontrollable malabsorption, we recommend home EN or PN for suitable patients.	Low/Strong Consensus
Role of immunonutrition/strong	In upper GI cancer patients undergoing surgical resection in the context of traditional perioperative care, we recommend oral/enteral immunonutrition.	High/Strong Consensus
